# Infected Spinal Hematoma After Accidental Exteriorization of an Epidural Catheter: A Case Report

**DOI:** 10.7759/cureus.47722

**Published:** 2023-10-26

**Authors:** Flávia Oliveira, Diana Fonseca, Mariana Fernandes, Ana Rita Teles, Sara Fonseca

**Affiliations:** 1 Department of Anesthesiology, Centro Hospitalar Universitário de São João, Porto, PRT

**Keywords:** conservative management, neurologic deficit, spinal epidural abscess, spinal epidural hematoma, epidural analgesia

## Abstract

Optimal treatment and outcome after spinal hematoma remain unclear. Close neurological monitoring is the key to successful treatment. Here, we present a case of an infected spinal hematoma management.

We report the case of a 51-year-old male patient, American Society of Anesthesiologists physical status I, admitted to a level I hospital due to right lower limb necrotizing fasciitis. A lumbar epidural catheter was placed for pain control without complications. On the 26th day of hospitalization, three hours after the accidental exteriorization of the epidural catheter, the patient developed severe back pain not relieved by systemic analgesia. Prophylactic low-molecular-weight heparin had been administered less than six hours before. The patient had inflammatory signs and digital pressure pain at the catheter insertion site and a positive Brudzinski sign. Sensory-motor deficits were never felt. An urgent dorsolumbar MRI exhibited a significant hematic collection at the subdural and anterior epidural level, and an infected epidural hematoma was assumed. Empirical antibiotics and hourly monitoring of sensory-motor deficits, meningeal signs, and fever were initiated. The patient remained on absolute bed rest. Low-molecular-weight heparin was stopped. The pain disappeared on the third day after the MRI. *Citrobacter koseri* was isolated. A re-evaluation CT scan did not show spinal compression. The patient was discharged on the 27th day after an MRI in an asymptomatic condition and was referred to acute pain consultation.

Neurological deficits are usually expected at the time of spinal hematoma diagnosis. However, the classic triad of pain, sensory deficits, and motor deficits is only seen in less than half of patients. Our patient only developed severe lumbar pain. In selected cases, diagnostic MRI associated with tight monitoring and conservative management can be applied.

## Introduction

Nowadays, the incidence of major complications after neuraxial anesthesia, such as spinal hematoma or abscess, in non-obstetric patients may range from 1:6,000 to as high as 1:1,000 epidural procedures. Therefore, it is more common than estimated in the past decades [1].

Due to the rare occurrence of these complications, literature is sparse regarding accurate data on risk factors, clinical courses, and recommendations for management strategies. Nevertheless, it appears that spinal hematomas are more likely to occur in older patients after epidural anesthesia and the first symptoms usually arise after catheter removal [1]. Furthermore, patients with higher American Society of Anesthesiologists physical status, a bloody tap during the procedure, and with sensory deficits before treatment appear to be at increased risk of persistent neurological deficits. Spinal abscess usually occurs after treatment with continuous epidural anesthesia and first symptoms often appear after spinal or epidural catheter removal [1].

In patients with mild neurological symptoms, a positive neurological outcome has been reported more frequently after conservative treatment than after surgical decompression [1,2]. In addition to the type of management, the interval between symptom onset and surgical intervention is also associated with outcome [1,3]. Close monitoring with regular neurological assessments seems to be crucial to determining patients who are suitable for conservative treatment and patients who should undergo surgery to avoid a potential worsening of symptoms [1].

Here, we report a case of an infected spinal hematoma, developed after the accidental removal of an epidural catheter, and its successful conservative treatment approach.

## Case presentation

A 51-year-old male patient, with a past medical history of urticaria, without usual medication or known drug allergies, was admitted to the emergency room due to necrotizing fasciitis of the right lower limb.

The patient had undergone multiple surgical interventions for debridement of devitalized soft tissues, with the first intervention occurring 24 hours after admission. For postoperative pain management, an opioid-sparing strategy was implemented, with lumbar epidural catheter placement on the eighth postoperative day in the intensive care unit (ICU). The technique was performed at L3-L4 level without complications (median approach, loss of resistance at 4.5 cm, 4 cm of catheter inserted, cephalic direction, one attempt).

The patient was evaluated daily by the Acute Pain Unit (APU) team. During this period, pain control was achieved and sensory-motor deficits (SMDs), dorsolumbar pain, or meningeal signs were never reported.

During hospitalization in the ICU, a diagnosis of bacteriemia by methicillin-resistant *Staphylococcus aureus* was made. Antibiotic therapy with clindamycin and daptomycin was instituted and the patient presented a favorable clinical course. Two weeks after the end of antibiotic therapy, the patient developed a fever with an increase in inflammatory parameters. Because of the presence of pain and inflammatory signs at the central venous catheter (CVC) insertion site, the CVC was removed and empiric antibiotherapy with daptomycin was instituted.

Three days later, urgent evaluation by the APU was requested due to the sudden onset of severe back pain just a few hours after the accidental exteriorization of the epidural catheter (day 26 after its placement). The patient was under prophylactic low-molecular-weight heparin (LMWH), with the last administration having occurred less than six hours before. On clinical and physical examination, the patient presented severe back pain, not relieved by systemic analgesia, inflammatory signs in the epidural catheter insertion area, and neck stiffness (negative Kernig sign with positive Brudzinski sign). No SMDs were noted. Systemic analgesia was adjusted to 1 g of paracetamol every six hours, tramadol perfusion (300 mg per day), and morphine SOS.

Due to suspicion of an epidural abscess with meningeal involvement, an urgent dorsolumbar MRI was requested, along with an Infectiology and Neurosurgery consultation. The antibiotic was changed to cefepime and vancomycin in meningeal doses and blood cultures were collected. Absolute bed rest was indicated, along with monitoring of SMD appearance, meningeal signs, and fever. The patient was informed of the alarm signs.

**Figure 1:**
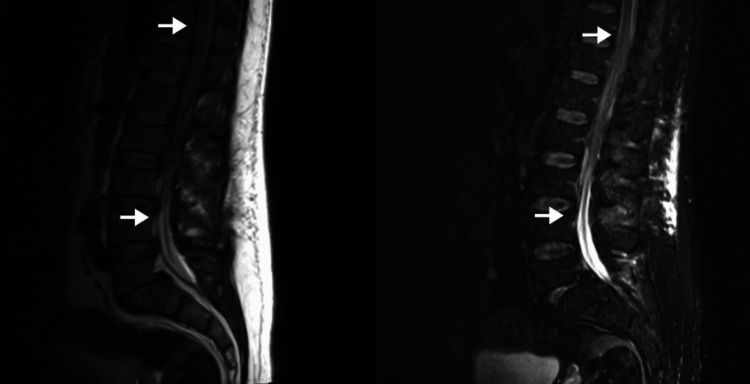
Patient’s dorsolumbar MRI showing a significant hematic collection at the subdural and anterior epidural level from D11 to L3. The arrows are delimiting the extent of the hematic collection. The top arrows show the superior part of the hematoma at the D11 level and the bottom arrows show the inferior part of the hematoma at the L3 level.

The MRI (Figure 1) showed a significant hematic collection at the subdural and anterior epidural level from D11 to L3, with linear uptake at the periphery and without areas of diffusion restriction. Prophylactic hypocoagulation was suspended and neurological status surveillance was maintained along with conservative treatment. The result of blood cultures was negative, but *Citrobacter koseri* was isolated at the tip of the epidural catheter, and antibiotherapy with cefepime was maintained for six weeks.

A dorsolumbar CT scan was performed 17 days after the dorsolumbar MRI, which did not identify intracanal blood collections with compressive effect. At this point, the patient started a functional rehabilitation program. During this period, the patient never experienced neurological deficits, and gradual improvement of inflammatory parameters was observed. The back pain disappeared on the third day after the MRI.

At the time of discharge from APU, the patient presented satisfactory lower limb pain control with paracetamol 1 g every six hours, tramadol 100 mg every eight hours, morphine 5 mg in SOS regimen, gabapentin 100 mg three times a day, and amitriptyline 25 mg once a day. There was no need for rescue analgesia. Moreover, there were no meningeal signs, back pain, SMDs, and no side effects of the prescribed medication.

As the patient was a foreigner and had to return to his country, alarm signs were explained, and the importance of re-evaluation and follow-up was reinforced. Hospital discharge occurred after 58 days (27 days after the infected spinal hematoma diagnosis) and the patient was asymptomatic.

## Discussion

Necrotizing skin and soft-tissue infections are rare but potentially life-threatening and disabling and often require ICU admission [4]. The stay of patients in the ICU increases the risk of infectious complications but allows the highest level of monitoring, vigilance, and patient care.

Perioperative pain management plays a central role in functional recovery after important noxious stimuli/lesions. Despite their risks and complications, regional and neuraxial analgesia techniques are becoming the best option to achieve optimal analgesia, as a part of an opioid-sparing strategy, particularly in critical and severely injured patients.

A catheter-associated spinal epidural hematoma can occur during catheter placement or removal. According to the last joint European Society of Anaesthesiology and Intensive Care/European Society of Regional Anaesthesia guidelines for regional anesthesia in patients on antithrombotic drugs, the last administration of low doses of LMWH should occur at least 12 hours before neuraxial procedures [5]. Accidental catheter exteriorization less than six hours after prophylactic LMWH medication administration may have been the cause of epidural hematoma development in this case.

In the present case, symptoms only developed on the epidural catheter exteriorization day, three days after the onset of fever and increased inflammatory markers. The patient had no neurological symptoms or low back pain previously. We could hypothesize that blood emerging in a space already occupied by an abscessed collection ends up promoting compression and the appearance of neurological symptoms, such as lumbar pain and Brudzinski sign. Indeed, a subclinical infection of the epidural canal could already be present, a sequel to bacterial translocation in a septic patient or resulting from the technique, and when the blood started to accumulate in the canal, due to catheter accidental exteriorization during the LMWH therapeutic window, the patient became symptomatic.

According to recent literature, approximately 97% of patients with spinal hematoma have neurological deficits at the time of diagnosis [1]. The classic triad of pain, sensory deficits, and motor deficits is seen in less than half of the patients (approximately 46%). The presence of back pain with no motor or sensory deficits, as in this case, is uncommon (approximately 10% of patients) [1].

Most commonly, spinal hematoma is seen in the epidural space; it is rarely seen in the subdural space or subarachnoid space. The subdural and subarachnoid hematomas can cause neural compression comparable to or worse than epidural hematoma [6]. The mechanism of hematoma formation in the spinal subdural space remains unclear [6]. 

It is crucial to maintain a high index of suspicion so as not to delay or misdiagnose this serious and potentially devastating complication, even if only non-specific symptoms appear or other overlapping confounding diagnoses are present, such as concomitant infections. MRI is the imaging examination of choice for diagnosing epidural hematoma [7].

In selected cases, mainly those with or without mild neurological symptoms, initial conservative management combined with frequent monitoring of the neurological function can be applied.

This case is uncommon as it is a mixed subdural and epidural infected hematoma, with the development of symptoms after an accidental catheter exteriorization, that was successfully managed with a conservative approach.

## Conclusions

The incidence of major complications after neuraxial anesthesia, such as spinal hematoma or abscess, is more common than estimated. Neurological deficits are expected at the time of diagnosis but the classic triad of pain and SMDs is not always present. A prompt diagnosis is crucial to avoid irreversible consequences for the patient. An early MRI is the imaging examination of choice for diagnosis.

Conservative treatment may be an option for some patients, mainly those with or without mild neurological symptoms. An effective conservative treatment involves a close follow-up with frequent neurological examination and imaging. A multidisciplinary approach, with integration and collaboration between the various medical specialties, is also essential for successful treatment.
